# How far along the future path do individuals recognize the path for stepping on multiple footfall targets? A new evaluation method under virtual reality

**DOI:** 10.3389/fspor.2025.1526576

**Published:** 2025-03-19

**Authors:** Ryotaro Waki, Kazuyuki Sato, Junki Inoue, Minoru Yamada, Takahiro Higuchi

**Affiliations:** ^1^ Department of Health Promotion Science, Tokyo Metropolitan University, Tokyo, Japan; ^2^ Department of Rehabilitation, Nakagawanosato Rehabilitation Center for Children with Disabilities, Saitama, Japan; ^3^ Department for the Psychology of Human Movement and Sport, Institute of Sport Science, Friedrich Schiller University Jena, Jena, Germany; ^4^ Technology Development Laboratories, Sony Corporation, Tokyo, Japan; ^5^ Faculty of Human Sciences, University of Tsukuba, Tokyo, Japan

**Keywords:** virtual reality, walking, multi-target stepping, path recognition, visual information

## Abstract

**Introduction:**

The ability to visually recognize the path ahead during walking is essential for adjusting gait patterns in an anticipatory manner to mitigate perturbations induced by tripping. In this study, we aimed to develop a walking task within a virtual reality (VR) environment, termed the VR multi-target stepping (VR-MTS) task, as a method to evaluate the extent to which individuals can recognize the path ahead while continuously stepping on footfall targets.

**Methods:**

As an initial study for the development of the VR multi-target stepping (VR-MTS) task, we tested a sample of young individuals (8 males and 5 females, aged 26.2 ± 3.7 years). Participants donned a head-mounted display (HMD) and walked for a distance of 4 m, under four distinct conditions. Participants were instructed to step on squares of specific color and that participants were instructed to step onto white squares that had been of the footfall target color for all conditions. In three of these conditions, all three colored squares present in the same row—located either one, two, or three rows ahead of the participants—were programmed to change to white (i.e., N + 1, N + 2, and N + 3 conditions). This setup was designed to evaluate the participant's ability to recognize the colors of the footfall targets at varying distances. In the control condition, no changes occurred in the colored squares during the walking task.

**Results and discussion:**

The rate of stepping failure was significantly higher under the N + 3 condition compared to the other three conditions. This finding suggests that young individuals are capable of recognizing footfall targets approximately two rows ahead when performing the VR multi-target stepping (VR-MTS) task but encounter difficulties when attempting to recognize targets located three rows ahead. Under the N + 3 condition, participants frequently stepped onto distractor squares, indicating a failure to recognize the stepping target situated three rows ahead, resulting in a random selection of the square to step on. Based on these findings, we conclude that the VR-MTS task is a valid method for evaluating visual recognition of the future path while stepping on multiple footfall targets.

## 1 Introduction

Visually recognizing the path ahead while walking is crucial for proactively adjusting gait patterns to prevent perturbation caused by tripping over obstacles (1–6). Moraes et al. illustrated that foot placement for stepping over obstacles begins several steps before reaching them during obstacle avoidance (3). Additionally, Krell et al. indicated that when individuals encounter two consecutive obstacles, they identify the position of a second step before negotiating the first (6). This insight highlights that visual information about an obstacle is obtained well before an individual approaches it.

Measurement of gaze behavior has revealed that some older adults experience challenges in utilizing visual information at a distance in a feedforward manner. One prominent behavioral characteristic contributing to this difficulty is a downward gaze during walking (7–9). When avoiding obstacles in their path, younger individuals typically direct their gaze toward the obstacle from a distance, whereas older adults more frequently focus their gaze on the ground near their feet (7). In our prior study, we measured gaze behavior as older participants performed a task known as the Multi-Target Stepping (MTS) task (10). This task required both older and younger participants to walk while stepping on target-colored squares (footfall targets) arranged across 15 rows, avoiding other-colored squares. The results indicated that younger participants fixated on the footfall target approximately three rows ahead, while older participants primarily fixated on a location closer to their imminent footfall target. This tendency was more pronounced in older participants at a higher risk of falling compared to those at lower risk. Additionally, these fixation patterns were significantly correlated with an increase in failures to step accurately on footfall targets; participants who directed their gaze closer to their feet exhibited more frequent stepping failures. These findings align with prior studies showing that, whereas younger individuals utilize visual information about an upcoming footfall target in a feedforward manner, older adults tend to rely on an online, feedback-driven approach (11, 12). Taken together, these results suggest that the downward gaze observed during walking indicates that some older individuals may face challenges in using visual information at a distance in a feedforward manner.

Several factors may contribute to the downward gaze in older adults. One key reason is the strategy of compensating for instability during standing and walking by fixating gaze on the ground (13, 14). Some studies showed that variability in postural sway is reduced when individuals look at nearby targets as opposed to distant targets (15–17). Older adults may try to take advantage of this effect by walking while gaze on the ground. Another contributing factor could be the difficulty encountered when walking under dual-task conditions (18–21). Studies have shown that during dual-task walking when older individuals perform a cognitive task such as memorizing words simultaneously, there is a significant decline in cognitive task performance. This suggests that maintaining balance during walking is more cognitively demanding for older individuals compared to younger ones. In essence, variability of postural sway can be modulated adaptively so as to facilitate the concurrent cognitive action, which is referred to as “supra-modal activity” (17). For example, Stoffregen et al. demonstrated that, when young individuals were tested, postural sway was reduced when they concurrently performed a task requiring precise control of eye movements, such as visual search of a target letter. This suggests that postural control and performing a cognitive task is not necessarily independent and that postural control can be modulated to improve cognitive performance. The lack of similar findings in older adults, as well as several reports showing impaired cognitive performance while maintaining balance (18–21), may result from their instability. Taken collectively, these findings imply that older adults may prioritize immediate balance maintenance, which can make it challenging for them to recognize distant paths in a feedforward manner (10).

A limitation of assessing visual recognition of the future path based on gaze behavior measurement is that, while these measurements indicate fixated locations, they do not necessarily correspond to the locations being actively recognized. This discrepancy arises because even when our gaze is fixed on a fixed point, we can still perceive the surroundings through peripheral vision (22, 23). Indeed, maintaining fixation on a specific location can sometimes function as a “visual pivot” (24, 25), allowing the surrounding area to be captured in the peripheral field of vision (26). Therefore, exploring alternative methods to determine how far ahead an individual recognizes their path while walking is necessary.

In this study, we aimed to develop a walking task in a VR environment to evaluate how far away an individual recognizes the future path when stepping on multiple footfall targets. Only young individuals were included, as the primary objective was to develop and validate the task. Previous study suggested that adjustments to footfall targets under a VR environment are similar in both younger and older adults (27). However, previous findings demonstrated changes in walking behavior under a VR environment, such as decreased walking speed or gait variability, particularly for younger adults (28, 29, 30), testing a new walking task in a VR environment with participants of younger adults would be useful to understand task characteristics. We reproduced the MTS task in a VR environment (VR-MTS task) (10, 31, 32), incorporating several modifications, such as extending the walking path to (4 m). Reproducing the task in a VR facilitated manipulation of experimental conditions, such as the positioning and visibility of footfall targets by turning all three colored squares in the same row white. These squares were positioned either one, two, or three rows ahead of participants (referred to as N + 1, N + 2, and N + 3 conditions, respectively). Under these conditions, participants would fail to step on the footfall target if they did not recognize its location before it turned white. By measuring the rate of trials in which young participants missed stepping on the footfall target that turned white, researchers estimate how far ahead young individuals recognize their intended path when stepping on multiple footfall targets.

The present study proposed two hypotheses. First, it was hypothesized younger participants would fail to step on the footfall target when the colored squares located three rows ahead turned white (i.e., under the N + 3 condition). Our previous study using the original MTS task demonstrated that young participants typically fixated on footfall targets positioned three rows ahead (10). However, preliminary findings indicated that the head flexion angle was slightly but significantly greater (i.e., participants looked more downward while walking) in the VR-MTS task compared to the original MTS task (See Appendix A for the detail). Based on this preliminary result, we hypothesized that participants in the VR-MTS task would have difficulty recognizing the footfall target location under the N + 3 condition. The Second hypothesis was that the greater the participant's head tilts downward while walking, the higher the rate of stepping failure would be. This hypothesis was grounded in the observation that individuals experience increased difficulty in viewing distant objects when their gaze is directed downward.

## 2 Materials and methods

### 2.1 Participants

Thirteen young individuals participated in the study (8 males and 5 females; mean age = 26.2 years, SD = 3.7 years). We verified, on a self-reported basis, that all participants had normal or corrected-to-normal vision, no current musculoskeletal injuries, and no neurological disorders. The mean standing height of participants was 169.2 cm (SD = 11.8 cm). The study was approved by the Ethics Committee of Tokyo Metropolitan University, Japan (approval number: H5–144). Written informed consent was obtained from all participants in accordance with the guidelines established by the Ethics Committee of Tokyo Metropolitan University and the Declaration of Helsinki.

### 2.2 Apparatus

The experiment was conducted in a room measuring 6.6 m × 5.6 m. Participants walked a distance of 4 m from the starting line along a walkway that was 5.5 m long and 1.25 m wide. A desktop computer (OMEN, HP Obelisk Desktop 875-1xxx, HP, USA) was utilized for data collection and stimulus presentation. Participants wore a HMD (Meta Quest 2, Meta, USA) with a resolution of 1,832 × 1,920 pixels per eye and a diagonal viewing angle of 90 degrees. The HMD was connected via a 5 m wired cable to ensure stable communication with the computer. Eighteen cameras were employed for motion analysis (Oqus300SYS or MIQUS, Qualisys, Sweden), tracking the spatial locations of passive retro-reflective markers at a sampling frequency of 90 Hz, which were processed using motion capture software (Qualisys Track Manager, Qualisys, Sweden). These cameras monitored a total of twenty-five markers to assess the location of the HMD, the floor, and the participant's entire body. Six markers were attached to the HMD, one of which was positioned on the forehead to measure head flexion angle. Two markers were affixed to the edges of the start and goal lines of the walkway. Additionally, regarding the markers placed on each participant's body, a single marker was attached to the lower jaw, five markers were placed on the dorsum of each foot to create a rigid body, and one marker each was attached to the left and right fifth metatarsal bones and heels. Three-dimensional marker positions were streamed from the motion analysis software (Qualisys Track Manager, Qualisys, Sweden) to the Unity game engine (Unity Technologies, USA) with a delay of approximately 50–70 ms. A video camera was employed to record the participants’ walking paths in the real environment, while the software “OBS Studio (OBS Project)” was utilized to capture the walking paths in the VR environment.

**Figure 1:**
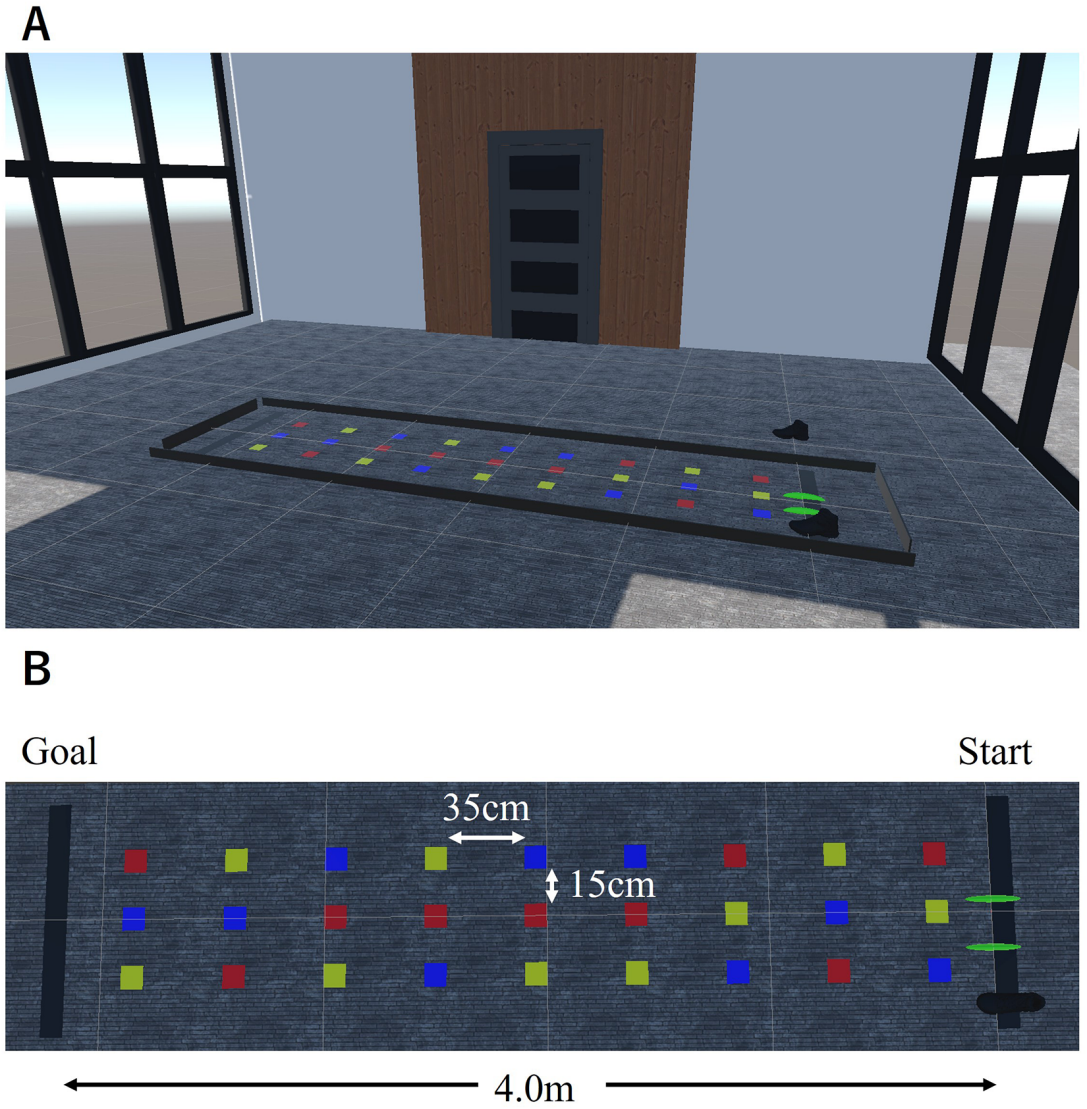
**(A)** Walkway and **(B)** placement of colored squares in the VR-MTS task.

Figure 1 shows the walkway used in the VR-MTS task. The walkway measured 4.0 m long by 1.0 m wide. It consisted of nine rows, each containing three 10 cm × 10 cm squares in three colors (red, blue, and yellow). Notably, the length of the walkway was shorter in the VR-MTS task than in the original MTS task (4 m vs. 10 m) due to laboratory constraints. Consequently, the distance between the squares in the two rows was also reduced (35 cm vs. 61 cm) to accommodate an increased number of rows of squares within the 4 m distance. The length of the walkway in this task, specifically 4 m, was determined based on the Short Physical Performance Battery (SPPB), which aims to provide a simple assessment of lower limb function in older adults (33). We conducted a preliminary experiment with this task configuration and confirmed that similar to the original MTS task, young participants experienced no stepping failures. This suggests that the VR-MTS task setting did not adversely affect their walking performance.

**Figure 2:**
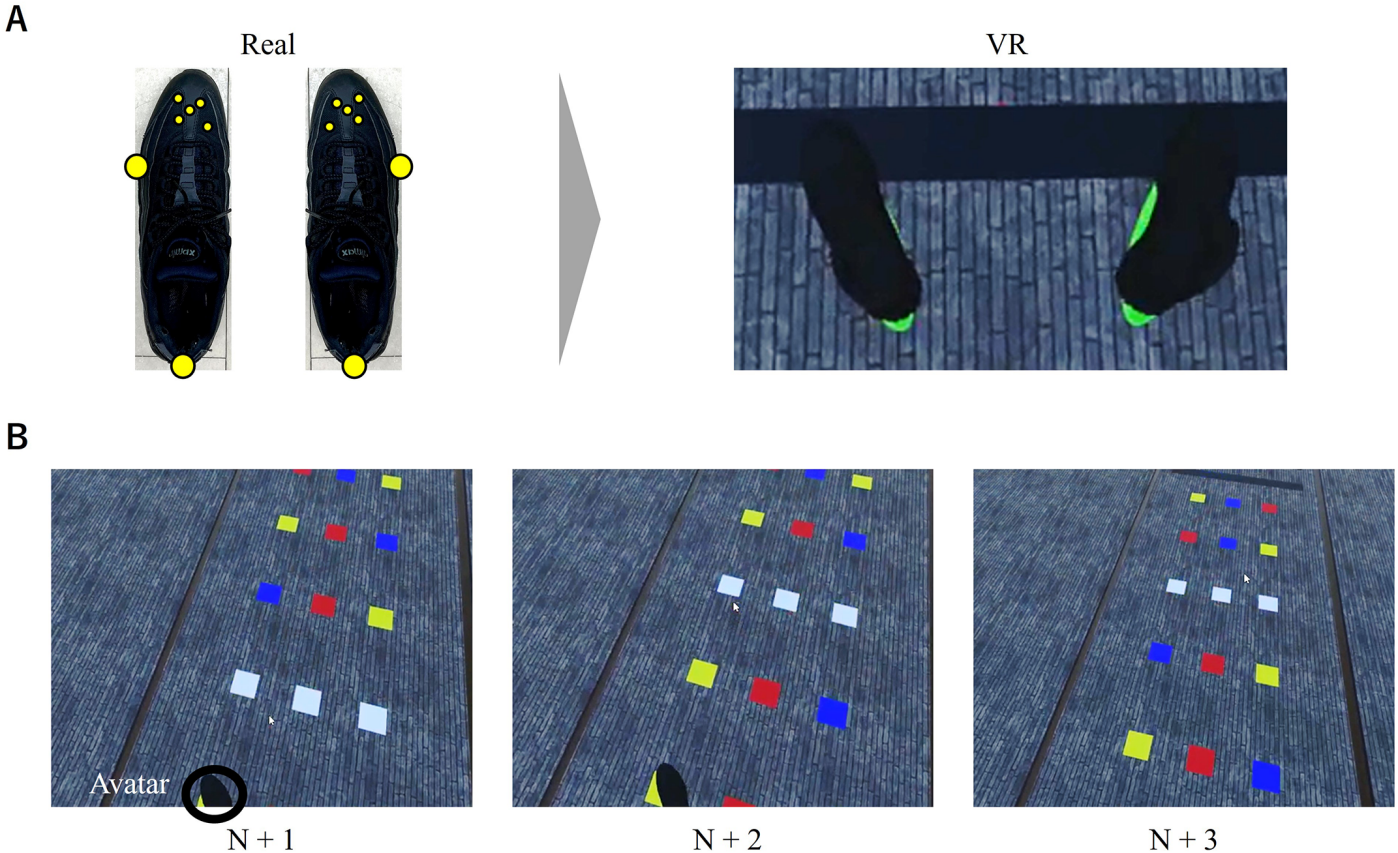
**(A)** Placement of reflexive markers on shoes (left) and the virtual avatar of shoes in VR (right), and **(B)** three experimental conditions in which the squares became white.

A virtual avatar of shoes (30 cm in length × 10 cm in width) was presented in synchronization with the location of a rigid body attached to the left and right dorsum of the foot (see Figure 2A). We chose to replicate only the foot position rather than the entire body position to prevent computer overload. We believe that presenting the shoe avatar was beneficial for task performance based on prior findings indicating that participants experienced a sense of body ownership and a walking sensation even when only the foot avatar was projected (34, 35).

### 2.3 Task and procedure

In the VR-MTS task, participants wearing the HMD walked a distance of 4 m and stepped on nine footfall targets. The arrangement of the colored squares was identical for all trials, as shown in Figure 1B. One of the three colors (i.e., red, blue, and yellow) was pre-specified as the footfall target to step on, while the others served as distractors that participants aimed to avoid. The color of the footfall target changed between trials. Prior to the start of each trial, participants stood at the start line indicated by a black line and waited for a verbal cue from the experimenter to begin the trial by closing their eyes. An experimenter informed the participant of the color of the footfall target to step onto before providing verbal instruction to start the trial. Participants opened their eyes and initiated walking at a comfortable speed. They were allowed to step on areas without colored squares if necessary. Participants stopped walking upon reaching the black line, which indicated where to stop. Due to technical issues with our experimental setup, participants removed the HMD after each trial and returned to the starting line. Feedback regarding stepping failures was provided during practice trials; however, no feedback was given for the main trials.

The task was conducted under four experimental conditions. In three of these conditions, three colored squares in the same row turned white when the participant's shoe avatar made contact with the colored squares in the fourth row from the start line. The three conditions varied in the distance of the white squares from the participants: one, two, or three rows ahead (referred to as N + 1, N + 2, and N + 3 conditions, respectively; see Figure 2B). When participants did not recognize which of the three white squares was the designated footfall target, they were instructed to select the one they believed to be the designated footfall target and stepped on it. In the control condition, there were no changes in the colored squares during walking. Participants were informed that the footfall targets would turn white during walking and that the order of all trials would be randomized before the intervention began. The participants were not informed of the number of squares ahead that would disappear before they initiated walking. Participants performed nine trials under each experimental condition, resulting in a total of 36 trials. To familiarize participants with the task, they completed a single practice trial under each of the four experimental conditions. The order of the practice trials was consistent for all participants: control, N + 1, N + 2, and N + 3.

### 2.4 Data analysis

We measured five dependent variables to evaluate participant performance in the VR-MTS task: rate of stepping failure, type of stepping failure, time required to complete the task (in seconds), number of steps taken to complete the task, and head flexion angle (in degrees). Stepping failure occurred when the avatar did not step on at least one of the nine footfall targets. The stepping failure included both (a) the participant did not step on a visibly colored footfall target and (b) the participant did not select the correct footfall target after its color changed to white. We calculated the proportion of participants (i.e., out of 13 participants) who experienced stepping failure at least once under each experimental condition. When a stepping failure occurred, we identified whether the participant stepped on a location other than the three squares (inaccurate step) or whether the participant failed to recognize the footfall target and stepped on a distractor (misrecognition). We then counted the number of participants who experienced each type of stepping failure. The time required to complete the task was defined as the difference between the time when the marker on the fifth metatarsal bone of the leading limb crossed the start line and when the same marker crossed the goal line. The number of steps taken to complete the task was counted as the number of times participants’ heels touched the floor during each trial. Head flexion angle was measured as an indicator of downward head tilting during walking. Since we could not measure gaze behavior in our setting, we used head flexion angle to estimate whether participants tended to look down. Positive values of the angle indicate a downward orientation. We followed the methodology outlined in a previous study (22) for calculating the head flexion angle. Specifically, we calculated the angle between a vertical line (parallel to the vertical axis) and a line connecting the forehead (a single marker from the HMD) to the lower jaw markers. The mean, maximum, and minimum values of the angle, as well as the range of the angle (maximum value minus minimum value), were calculated. The maximum angle represented the extent to which the head was tilted during each trial, while the minimum angle indicated how far ahead the head was directed. The range of the angle reflects the extent of head movement during walking.

Cochran's *Q* test was employed to statistically test the rate of stepping failure, as the rate is on a nominal scale. For the type of stepping failure, McNemar's test was used to compare the rates of the two types. For other dependent variables, we compared the values among the four experimental conditions using a one-way analysis of variance (ANOVA). When a significant main effect was found, Bonferroni's *post-hoc* tests were conducted. Furthermore, we calculated Pearson's correlation coefficients between the number of stepping failures and the other dependent variables to discuss what influenced the quality of stepping performance. The correlation coefficient was calculated using the average number of stepping failures per trial, as the rate of stepping failure is a nominal measure and cannot be used to compute the correlation coefficient. Statistical analyses were performed using IBM Statistical Package for the Social Sciences (SPSS) for Windows, version 26 (IBM Corp., Armonk, NY, USA). Statistical significance was set at *p* < 0.05.

## 3 Results

**Figure 3:**
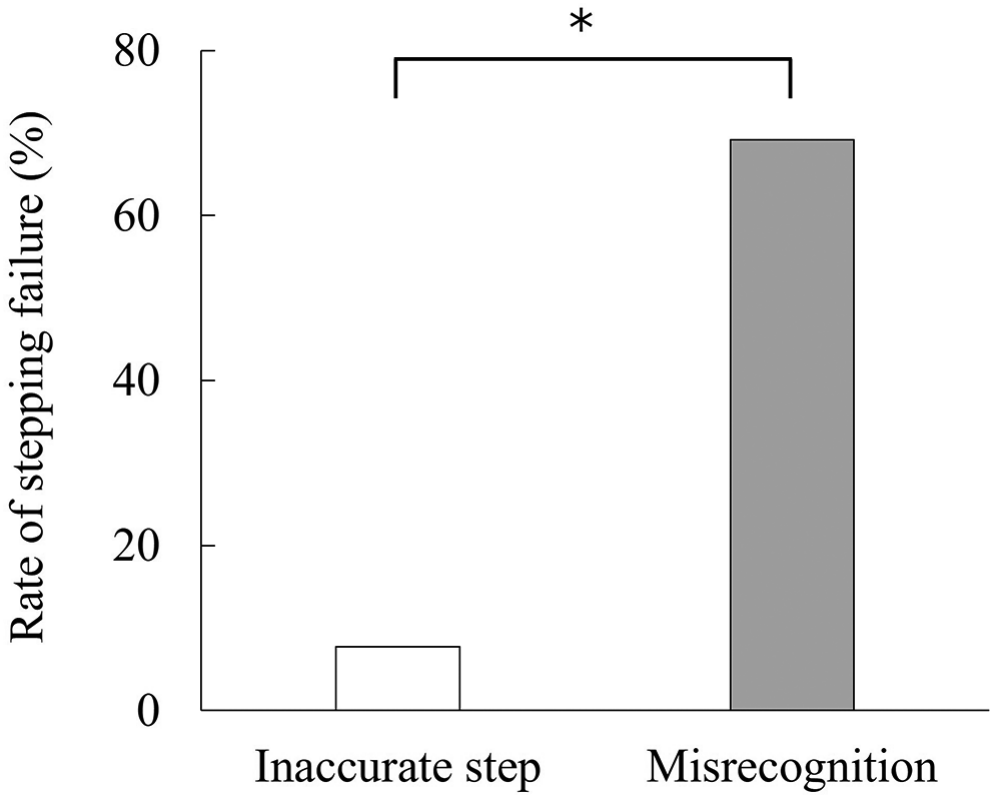
Rate of inaccurate step and misrecognition observed when stepping failure occurs. *Indicates a significant difference.

No stepping failures occurred under the control, N + 1, or N + 2 conditions (i.e., 0%). Stepping failures were observed only under the N + 3 condition [69.2%, Cochran's Q (3) = 27.000, *p* < 0.001]. Nine participants experienced a total of 15 stepping failures (Table 1). The types of stepping failures are depicted in Figure 3, indicating that these failures primarily resulted from misrecognition [McNemar's chi-squared (1) = 6.125, *p* = 0.008].

**Table 1:** Four dependent variables under each experimental condition.

Conditions	N + 1	N + 2	N + 3	Control	Statistics
Rate of stepping failure (%)^a^	0 (0)	0 (0)	9 (69.2)	0 (0)	3A > Co, N + 1, N + 2
Time required to complete the task (sec)^b^	5.60 ± 1.08	5.58 ± 1.08	5.57 ± 1.08	26.18 ± 7.18	No statistical comparisons were made
Number of steps taken to complete the task (steps)^b^	9.33 ± 0.31	9.31 ± 0.35	9.32 ± 0.35	9.36 ± 0.40	No statistical comparisons were made
Mean value of head flexion angle (deg)^b^	51.52 ± 5.23	51.89 ± 5.55	51.58 ± 5.40	51.44 ± 5.38	No statistical comparisons were made
Maximum value of head flexion angle (deg)^b^	59.58 ± 5.19	60.20 ± 5.31	59.86 ± 4.72	59.90 ± 4.98	No statistical comparisons were made
Minimum value of head flexion angle (deg)^b^	44.12 ± 5.88	44.40 ± 6.09	44.34 ± 5.74	43.92 ± 6.09	No statistical comparisons were made
Range of angle of head flexion angle (deg)^b^	15.46 ± 2.19	15.80 ± 2.79	15.52 ± 2.46	15.98 ± 2.65	No statistical comparisons were made

^a^ Cochran's *Q* test, number (%).

^b^ A one-way ANOVA, mean ± standard deviation.

The time required to complete the task and the number of steps taken to complete the task under each experimental condition are presented in Table 1. No significant main effects were found for both dependent variables [F(3,36) = 2.141, *p* = 0.112, *η*p^2^ = 0.151; and F(3,36) = 0.646, *p* = 0.591, *η*p^2^ = 0.051, respectively]. The mean, maximum, and minimum head flexion angles for each experimental condition are also listed in Table 1. There were no significant main effects on these three variables [mean value: F(3,36) = 0.811, *p* = 0.496, *η*p^2^ = 0.063; maximum value: F(3,36) = 1.029, *p* = 0.391, *η*p2 = 0.079; and minimum value: F(3,36) = 0.789, *p* = 0.508, *η*p^2^ = 0.062].

The correlation analysis between the number of stepping failures and other dependent variables is presented in Table 2. The angle range of head flexion angle was negatively correlated with the number of stepping failures (*r* = −0.597, *p* = 0.031). No other significant correlations were found.

**Table 2:** Statistical data (*r* and *p*-value) of Pearson's correlation coefficient between the number of stepping failures and other measurements.

Measurement	*r*	*p*
Time required to complete the task (sec)	−0.174	0.569
Number of steps taken to complete the task (steps)	0.321	0.285
Mean value of head flexion angle (deg)	0.159	0.604
Maximum value of head flexion angle (deg)	0.017	0.957
Minimum value of head flexion angle (deg)	0.254	0.402
Angle range of head flexion angle (deg)	−0.597	0.031*

* *p* < 0.05.

## 4 Discussion

In this study, we examined whether manipulating the visibility of a footfall target using the VR-MTS task is an effective method for evaluating how far ahead an individual can recognize targets while walking. Participants demonstrated a significantly greater rate of stepping failure under the N + 3 condition, in which the footfall target three rows ahead turned white (Table 1). The stepping failures primarily resulted from misrecognition rather than inaccurate stepping, indicating that participants did not recognize the stepping target located three rows ahead and, consequently, randomly chose a square to step on (Figure 3). These results support our first hypothesis, suggesting that manipulating the visibility of the footfall target in the VR-MTS task increases the rate of stepping failures three rows ahead due to difficulties in using visual information at a distance.

The correlation analyses revealed that contrary to our second hypothesis, there was no significant correlation between the number of stepping failures and either the mean or maximum value of the head flexion angle. Instead, a significant negative correlation was found between the number of stepping failures and the range of head flexion angle (Table 2). This indicates that the narrower the range of head movement while performing the task, the greater the number of stepping failures. In other words, although head movement and task performance were related, it was the narrow range of head movement—rather than downward head tilting—that negatively impacted task performance.

The present findings generally support the conclusion that the VR-MTS task is a valid method for evaluating visual recognition of the future path when stepping on multiple footfall targets. Until now, the primary method for assessing how far ahead individuals recognize targets during walking has been to make inferences based on gaze behavior. By utilizing the VR-MTS task, we can accurately quantify how many steps ahead an individual is looking while walking to step on multiple footfall targets. A previous study using the MTS task to measure gaze behavior indicated that younger participants fixated on a footfall target located approximately three rows ahead, whereas older participants focused on locations closer to the imminent footfall target (10). This tendency was particularly pronounced among older adults at high risk of falling. Future studies employing the VR-MTS task could more clearly elucidate the extent to which older adults have a reduced ability to recognize distant locations compared to younger adults.

Matthis et al. conducted a series of obstacle avoidance tasks by manipulating the number of steps ahead so that participants could see the obstacle while walking. They demonstrated that, for young adults, visual information at least two steps ahead was necessary for stepping on multiple footfall targets (36). Our findings are consistent with those of Matthis et al. in that visual information two rows ahead was recognized, which is virtually equivalent to two steps ahead due to the short distance between the rows of squares. A novel finding of the present study is that participants did not seem to recognize locations further away; specifically, young participants failed to recognize three rows ahead for path planning. This suggests that, when planning for multiple footfall targets, the distance individuals can visually recognize is relatively short, even though the upper peripheral vision covers a greater distance.

The narrower range of head movement during the VR-MTS task, rather than downward head tilting, was correlated with the stepping failure. One possible explanation for this outcome is that the VR-MTS task necessitates two types of behavioral adjustments: looking forward to recognizing the path ahead and looking downward to accurately step on the target. For advanced recognition of the path, the gaze is generally directed several steps ahead (36, 37). However, on complex terrain, the gaze often shifts toward the feet to ensure accurate foot placement (38). A greater range of head movement might have been beneficial for successfully performing the VR-MTS task, as it would support both forward path recognition and accurate stepping on the target. Future measurements of eye-tracking data in addition to the range of head movement would be beneficial to examine whether this assumption holds true.

The average head flexion angle while performing the VR-MTS task was 51.60 ± 5.15 deg. This angle was approximately 10° smaller than that recorded in the preliminary experiment (59.53 ± 6.72 deg), where colored squares on the ground were always visible, and there was no manipulation of squares turning white (see Appendix A). Notably, no significant differences were observed for any other dependent measures (e.g., the time required to complete the task and the number of steps taken to complete the task). These findings suggest that participants adjusted their gaze forward to adapt to the manipulation of footfall targets in the VR-MTS task. This adaptation aligns with the experimental requirement to quickly recognize the color of a distant target before it disappears. Leveraging this property, the VR-MTS task could serve not only as an assessment tool for visual recognition but also as an intervention to encourage participant to direct their gaze further ahead. Previous studies have shown that performance in stepping on multiple footfall targets improves when the gaze is more prospective (32, 39). Future research should explore whether repeated exposure to the VR-MTS task can guide older adults to shift their gaze to a greater distance.

This study has several limitations. First, the small sample size restricts the conclusions that can be drawn. Second, the placement of colored squares was identical across all trials. Although this consistency was necessary for reasons to avoid potential cognitive loads and kinematic loads resulted from walking while navigating a complex sequence of multiple turning for each trial that could arise when a randomized arrangement of the colored squares across trials was used, it may have allowed participants to memorize the placement of the colored squares in later trials. One might assume that participants could have performed the task without missing the footfall target, even if forward path recognition was challenging. To examine the impact of this issue, we analyzed the rate of stepping failures in the latter 18 trials out of a total of 36 trials for each participant. The results indicated that six of the nine participants exhibited stepping failures in the latter 18 trials. This finding suggests that the memorization of the placement of the colored squares did not significantly affect performance; however, this issue should be investigated in more detail by randomly varying the placement of squares in each trial. Third, the stepping failures observed in the present study could occur not only when the future path is unrecognized but also when the future path is recognized but subsequently forgotten during walking. To establish that the stepping failures were indeed caused by a lack of recognition of the future path, measuring eye movement would be beneficial. If an individual recognizes the future path in the VR-MTS task but forgets to do so during walking, the individual would likely gaze at the footfall target at least once before it is concealed. Future evaluations of gaze behavior while performing the VR-MTS task should be conducted to explore this aspect further. Notably, evaluations of gaze behavior is also helpful to discuss more deeply about the meaning of looking the path ahead during walking while continuously stepping on footfall targets. As discussed, performance in stepping on multiple footfall targets may improve when the gaze is more prospective (32, 39). However, Stoffregen et al. demonstrated that it is the direction of looking (i.e., looking forward or looking down), rather than the physical distance of the visible surrounding that affects variability in postural sway (17). That is, variability in postural sway may be greater when individuals look at a distance than when they look down. Measuring eye movement while experimentally manipulating how far ahead participants are looking while performing the VR-MTS task will inform whether walking with eyes fixed on the distance really improve performance of the task in older adults beyond the concern of increased postural sway.

In conclusion, this study indicates that the VR-MTS task effectively demonstrates how far ahead younger individuals can recognize targets while walking and stepping onto multiple footfall targets. The VR-MTS task developed in this study suggests the potential for evaluating path recognition in greater detail, a concept that has previously been discussed at a speculative level based on gaze behavior. Future studies are necessary to examine whether such experimental manipulations can effectively evaluate the path recognition of individuals who have difficulty utilizing visual information at a distance in a feedforward manner, particularly among older adults.

## Data Availability

The raw data supporting the conclusions of this article will be made available by the authors, without undue reservation.

## Appendix A

We conducted a preliminary experiment to verify that there were no critical differences in performance (e.g., the rate of stepping failure) between the VR-MTS task in the current setting and the MTS task performed in a real environment. The settings of the VR-MTS task were identical to those used in this study. The MTS task performed in the real environment was adjusted so that the walking path and the locations of the colored squares matched those of the VR-MTS task. Additionally, the visibility of the footfall target was not manipulated during the VR-MTS task in the preliminary experiment. Fifteen young individuals (27.7 ± 4.1 years old) performed 15 trials for each task. The rate of stepping failure and head flexion angle across the 15 trials were measured. Stepping failure was defined as a failure to step on at least one of the nine footfall targets during a trial. The head flexion angle was measured as an indicator of downward head tilting while walking. Since direct gaze behavior could not be measured in the setting, head flexion angle served whether participants tended to look down. Positive values of the angle represented the extent of the head was tilt during trial. McNemar's test was conducted to analyze the rate of stepping failure, and a paired t-test was used to evaluate the difference in head flexion angle. The result showed no significant difference in the rate of stepping failure between the two tasks. However, the means e head flexion angle in the VR-MTS task was significantly higher than in the MTS task. This finding indicates that participants successfully adapted to the VR-MTS in the current experimental setup. Notably, the mean head flexion angle was slightly but significantly different between the two tasks (59.53 ± 6.72 deg for VR-MTS and 42.44 ± 7.79 deg for the MTS), suggesting that participants walked with their heads slightly more tilted downward when performing the VR-MTS.

-----------------------

## References

[1] Higuchi T. Visuomotor control of human adaptive locomotion: understanding the anticipatory nature. Front Psychol. (2013) 4:277. 10.3389/fpsyg.2013.00277 23720647 PMC3655271

[2] Hollands M Patla A Vickers J. “Look where you're going!”: gaze behavior associated with maintaining and changing the direction of locomotion. Exp Brain Res. (2002) 143(2):221–30. 10.1007/s00221-001-0983-7 11880898

[3] Moraes R Lewis MA Patla AE. Strategies and determinants for selection of alternate foot placement during human locomotion: influence of spatial and temporal constraints. Exp Brain Res. (2004) 159(1):1–13. 10.1007/s00221-004-1888-z 15448958

[4] Sreenivasa MN Frissen I Souman JL Ernst MO. Walking along curved paths of different angles: the relationship between head and trunk turning. Exp Brain Res. (2008) 191(3):313–20. 10.1007/s00221-008-1525-3 18688604

[5] Matthis JS Yates JL Hayhoe MM. Gaze and the control of foot placement when walking in natural terrain. Curr Biol. (2018) 28(8):1224–1233.e5. 10.1016/j.cub.2018.03.008 29657116 PMC5937949

[6] Krell J Patla AE. The influence of multiple obstacles in the travel path on avoidance strategy. Gait Posture. (2002) 16(1):15–9. 10.1016/s0966-6362(01)00194-1 12127182

[7] Paquette MR Vallis LA. Age-related kinematic changes in late visual-cueing during obstacle circumvention. Exp Brain Res. (2010) 203(3):563–74. 10.1007/s00221-010-2263-x 20467732

[8] Zito GA Cazzoli D Scheffler L Jäger M Müri RM Mosimann UP Street crossing behavior in younger and older pedestrians: an eye- and head-tracking study psychology, psychiatry and quality of life. BMC Geriat. (2015) 15:176. 10.1186/s12877-015-0175-0 PMC4696098 26714495

[9] Maslivec A Bampouras TM Dewhurst S. Head flexion and different walking speeds do not affect gait stability in older females. Hum Mov Sci. (2017) 55:87–93. 10.1016/j.humov.2017.08.001 28802896

[10] Yamada M Higuchi T Mori S Uemura K Nagai K Aoyama T Maladaptive turning and gaze behavior induces impaired stepping on multiple footfall targets during gait in older individuals who are at high risk of falling. Arch Gerontol Geriatr. (2012) 54(2):e102–8. 10.1016/j.archger.2011.08.012 21908059

[11] Patla AE Vickers JN. How far ahead do we look when required to step on specific locations in the travel path during locomotion? Exp Brain Res. (2003) 148(1):133–8. 10.1007/s00221-002-1246-y 12478404

[12] Chapman GJ Hollands MA. Age-related differences in stepping performance during step cycle-related removal of vision. Exp Brain Res. (2006) 174(4):613–21. 10.1007/s00221-006-0507-6 16733708

[13] Aoki O Otani Y Morishita S Domen K. The effects of various visual conditions on trunk control during ambulation in chronic post-stroke patients. Gait Posture. (2017) 52:301–7. 10.1016/j.gaitpost.2016.12.018 28033576

[14] Koren Y Handelzalts S Parmet Y Bar-Haim S. Older adults and stroke survivors are steadier when gazing down. PLoS One. (2023) 18(5):e0285361. 10.1371/journal.pone.0285361 37205706 PMC10198484

[15] Lee D Lishman J. Visual proprioceptive control of stance. J Hum Mov Stud. (1975) 1:87–95.

[16] Stoffregen TA Smart LJ Bardy BG Pagulayan RJ. Postural stabilization of looking. J Exp Psychol Hum Percept Perform. (1999) 25(6):1641–58. 10.1037/0096-1523.25.6.1641

[17] Stoffregen TA Hettinger LJ Haas MW Roe MM Smart LJ. Postural instability and motion sickness in a fixed-base flight simulator. Hum Factors. (2000) 42(3):458–69. 10.1518/001872000779698097 11132807

[18] Schaefer S Schellenbach M Lindenberger U Woollacott M. Walking in high-risk settings: do older adults still prioritize gait when distracted by a cognitive task? Exp Brain Res. (2015) 233(1):79–88. 10.1007/s00221-014-4093-8 25224704

[19] Verghese J Kuslansky G Holtzer R Katz M Xue X Buschke H Walking while talking: effect of task prioritization in the elderly. Arch Phys Med Rehabil. (2007) 88(1):50–3. 10.1016/j.apmr.2006 17207675 PMC1894901

[20] Li KZH Lindenberger U Freund AM Baltes PB. Walking while memorizing: age-related differences in compensatory behavior. Psychol Sci. (2001) 12(3):230–7. 10.1111/1467-9280.00341 11437306

[21] Lindenberger U Marsiske M Baltes PB. Memorizing while walking: increase in dual-task costs from young adulthood to old age. Psychol Aging. (2000) 15(3):417–36. 10.1037//0882-7974.15.3.417 11014706

[22] Marigold DS Patla AE. Visual information from the lower visual field is important for walking across multi-surface terrain. Exp Brain Res. (2008) 188(1):23–31. 10.1007/s00221-008-1335-7 18322679

[23] Feld JA Plummer P. Visual scanning behavior during distracted walking in healthy young adults. Gait Posture. (2019) 67:219–23. 10.1016/j.gaitpost.2018.10.017 30380505

[24] Ripoll H Kerlirzin Y Stein JF Reine B. Analysis of information processing, decision making, and visual strategies in complex problem-solving sport situations. Hum Mov Sci. (1995) 14(3):325–49. 10.1016/0167-9457(95)00019-O

[25] Williams AM Elliott D. Anxiety, expertise, and visual search strategy in karate. J Sport Exerc Psychol. (1999) 21(4):362–75. 10.1123/JSEP.21.4.362

[26] Cinelli ME Patla AE Allard F. Behaviour and gaze analyses during a goal-directed locomotor task. Q J Exp Psychol. (2009) 62(3):483–99. 10.1080/17470210802168583 18618377

[27] Delbes L Mascret N Goulon C Montagne G. Differences of gait adaptability behavior between young and healthy older adults during a locomotor pointing task in virtual reality. Gait Posture. (2024) 109(1):233–9. 10.1016/j.gaitpost.2024.02.009 38364510

[28] Janeh O Langbehn E Steinicke F Bruder G Gulberti A Poetter-Nerger M. Walking in virtual reality: effects of manipulated visual self-motion on walking biomechanics. ACM Trans Appl Percept. (2017) 14(2):1–15. 10.1145/3022731

[29] Janeh O Bruder G Steinicke F Gulberti A Poetter-Nerger M. Analyses of gait parameters of younger and older adults during (non-)isometric virtual walking. IEEE Trans Vis Comput Graph. (2018) 24(10):2663–74. 10.1109/TVCG.2017.2771520 29990158

[30] Horsak B Simonlehner M Schöffer L Dumphart B Jalaeefar A Husinsky M. Overground walking in a fully immersive virtual reality: a comprehensive study on the effects on full-body walking biomechanics. Front Bioeng Biotechnol. (2021) 9:780314. 10.3389/fbioe.2021.780314 34957075 PMC8693458

[31] Yamada M Higuchi T Tanaka B Nagai K Uemura K Aoyama T Measurements of stepping accuracy in a multi-target stepping task as a potential indicator of fall risk in elderly individuals. J Gerontol A Biol Sci Med Sci. (2011) 66(9):994–1000. 10.1093/gerona/glr073 21746737

[32] Yamada M Higuchi T Nishiguchi S Yoshimura K Kajiwara Y Aoyama T. Multi-target stepping program in combination with a standardized multicomponent exercise program can prevent falls in community-dwelling older adults: a randomized, controlled trial. J Am Geriatr Soc. (2013) 61(10):1669–75. 10.1111/jgs.12453 24001116

[33] Guralnik JM Simonsick EM Ferrucci L Glynn RJ Berkman LF Blazer DG A short physical performance battery assessing lower extremity function: association with self-reported disability and prediction of mortality and nursing home admission. J Gerontol. (1994) 49(2):M85–94. 10.1093/geronj/49.2.m85 8126356

[34] Kondo R Sugimoto M Minamizawa K Hoshi T Inami M Kitazaki M. Illusory body ownership of an invisible body interpolated between virtual hands and feet via visual-motor synchronicity. Sci Rep. (2018) 8(1):4–6. 10.1038/s41598-018-25951-2 29765152 PMC5954161

[35] Matsuda Y Nakamura J Amemiya T Ikei Y Kitazaki M. Enhancing virtual walking sensation using self-avatar in first-person perspective and foot vibrations. Front Virtual Real. (2021) 2:1–11. 10.3389/frvir.2021.654088

[36] Matthis JS Fajen BR. Visual control of foot placement when walking over complex terrain. J Exp Psychol Hum Percept Perform. (2014) 40(1):106–15. 10.1037/a0033101 23750964

[37] McFadyen BJ Fiset F Charette C. Substituting anticipatory locomotor adjustments online is time-constrained. Exp Brain Res. (2018) 236(7):1985–96. 10.1007/s00221-018-5277-4 29725704

[38] Bonnen K Matthis JS Gibaldi A Banks MS Levi DM Hayhoe M. Binocular vision and the control of foot placement during walking in natural terrain. Sci Rep. (2021) 11(1):20881. 10.1038/s41598-021-99846-0 34686759 PMC8536664

[39] Cates A Gordon KE. Don't watch your step: gaze behavior adapts to the practice of a target stepping task. J Neurophysiol. (2022) 128(3):445–54. 10.1152/jn.00155.2022 35822745 PMC9423783

